# 1,3,5-Tri­aza-7-phosphaadamantan-1-ium nitrate

**DOI:** 10.1107/S2414314626009296

**Published:** 2026-09-11

**Authors:** Adesola A. Adeleke, Sizwe J. Zamisa, Bernard Omondi, Olatunde S. Olatunji

**Affiliations:** a School of Agriculture and Science, Discipline of Chemistry, University of KwaZulu-Natal, Private Bag X54001, Durban, 4000, South Africa; b Department of Chemical Sciences, Olabisi Onabanjo University, Ago-Iwoye, Nigeria; Howard University, USA

**Keywords:** crystal structure, PTAH^+^, nitrate salt, supra­molecular network

## Abstract

The title salt, C_6_H_13_N_3_P^+^·NO_3_^−^ or [PTAH][NO_3_], crystallizes in the triclinic space group *P*1 with two independent PTAH^+^ cations and two nitrate anions in the asymmetric unit. In the crystal, N—H⋯O and C—H⋯O hydrogen bonds link the PTAH^+^ and oxygen atoms of the nitrate anion, forming a three-dimensional supra­molecular structure.

## Abstract 2

The title salt comprises a N-protonated PTA cation with a nitrate counter-ion.

## Graphical abstract

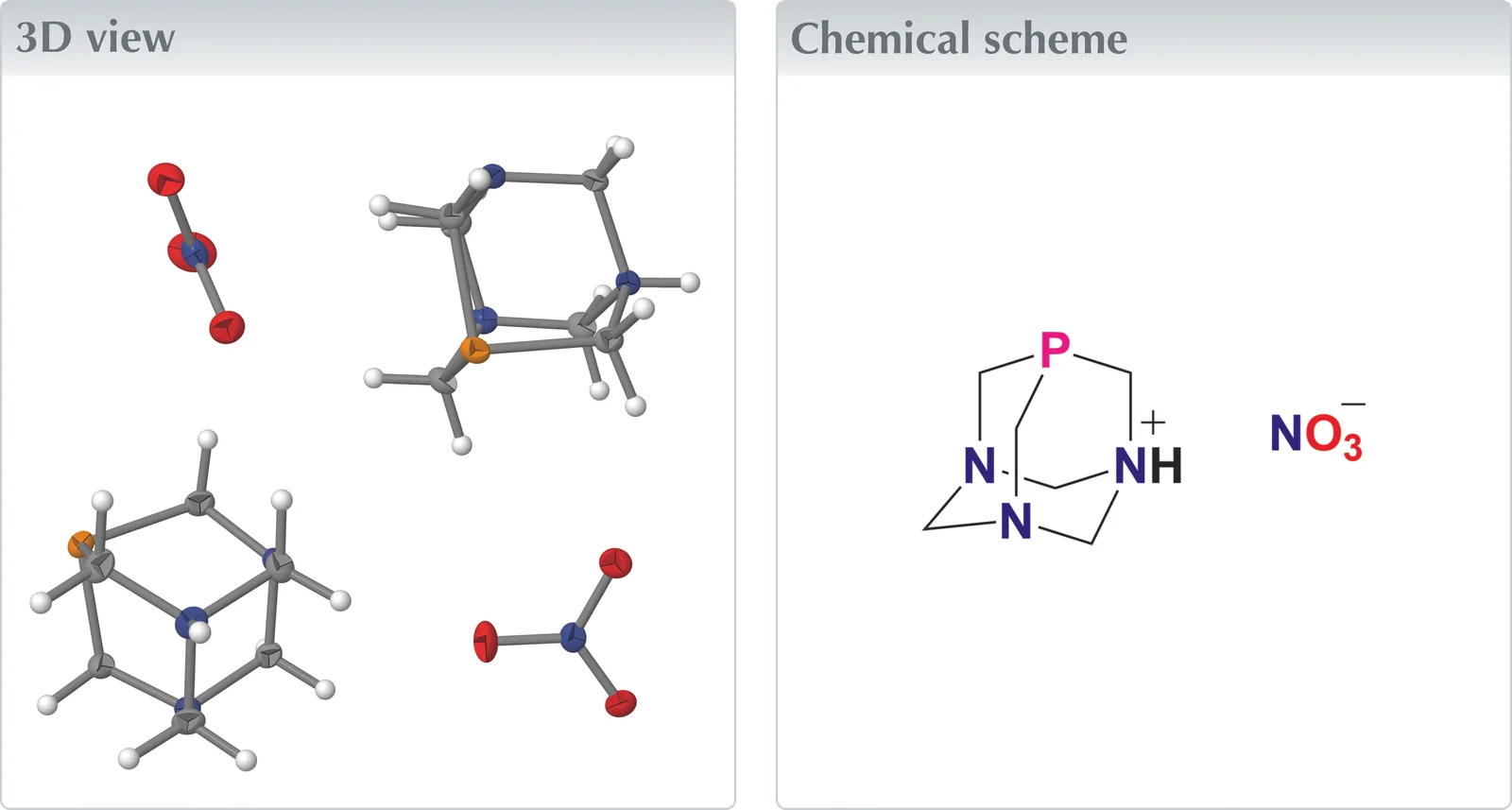

## Structure description

1,3,5-Tri­aza-7-phosphaadamantane (PTA) is a well established water-soluble cage-type phosphine that incorporates a soft phospho­rus donor atom at the bridgehead position 7 and three nitro­gen atoms at positions 1, 3, and 5 of an adamantane-like bicyclic framework (Daigle *et al.*, 1998 ▸). Protonation at one of these nitro­gen atoms readily yields the PTAH^+^ (C_6_H_13_N_3_P^+^) cation, which is commonly encountered during the synthesis of PTA-based metal–phosphine complexes (Kirillov *et al.*, 2007 ▸; Tu *et al.*, 2008 ▸).

The title compound shows solid-state structural features for a N– protonated PTAH^+^ cage in the absence of metal coordination. The asymmetric unit of the title compound contains two independent PTAH^+^ cations together with two nitrate anions (Fig. 1 ▸). The P—C bond distances in the PTA moieties are between 1.847 (4) and 1.856 (3) Å with C—P—C bond angles between 96.7 (1) and 96.8 (2)°. The N—C bond distances of the non-protonated nitro­gen atoms of both PTA moieties are between 1.434 (5)–1.482 (5) Å, while the N—C distances at the protonation nitro­gen centres are observed between 1.501 (4) and 1.524 (5) Å. The N—C distances at the protonated N atoms are notably longer than N—C distances of the non-protonated nitro­gen atoms of the PTA moieties, which are consistent with the expected weakening of N—C bonds upon quaternization of the nitro­gen atom. All distances and angles are in good agreement with those reported for structurally related PTA-based compounds (Akbayeva *et al.*, 2005 ▸; Kirillov *et al.*, 2007 ▸; Tu *et al.*, 2008 ▸). In the crystal, N—H⋯O, N—H⋯(O,O) and C—H⋯O hydrogen bonds (Table 1 ▸) link the PTAH^+^ NH groupings and oxygen atoms of the nitrate anion, forming a three-dimensional supra­molecular structure as depicted in Fig. 2 ▸.

## Synthesis and crystallization

PTA was prepared according to the literature method (Daigle *et al.*, 1998 ▸). In an attempt to synthesize a PTA–Zn^II^ complex, an aqueous solution of Zn(NO_3_)_2_·6H_2_O (0.297 g, 1.0 mmol) in distilled water (5 ml) was added to a solution of PTA (0.312 g, 2.0 mmol) in distilled water (10 ml) and the mixture was stirred at room temperature for 30 minutes. The colourless solution was filtered and the filtrate was transferred to an open vial and allowed to evaporate slowly at ambient temperature. After several days, colourless block-shaped crystals of the title compound, [PTAH]_2_[NO_3_]_2_, were isolated and identified by single-crystal X-ray diffraction analysis. The formation of the protonated phosphine salt rather than the intended Zn^II^ complex can be attributed to N-protonation of PTA in the weakly acidic nitrate medium, which is consistent with a related literature report (Akbayeva *et al.*, 2005 ▸).

## Refinement

Crystal data, data collection and structure refinement details are summarized in Table 2 ▸. The crystal chosen for data collection was found to be twinned and was modelled using twin law (1 0 0) with a BASF value of 0.0628 (6).

## Supplementary Material

Crystal structure: contains datablock(s) I. DOI: 10.1107/S2414314626009296/bv4062sup1.cif

Structure factors: contains datablock(s) I. DOI: 10.1107/S2414314626009296/bv4062Isup2.hkl

Supporting information file. DOI: 10.1107/S2414314626009296/bv4062Isup3.cml

CCDC reference: 2585607

Additional supporting information:  crystallographic information; 3D view; checkCIF report

## Figures and Tables

**Figure 1:**
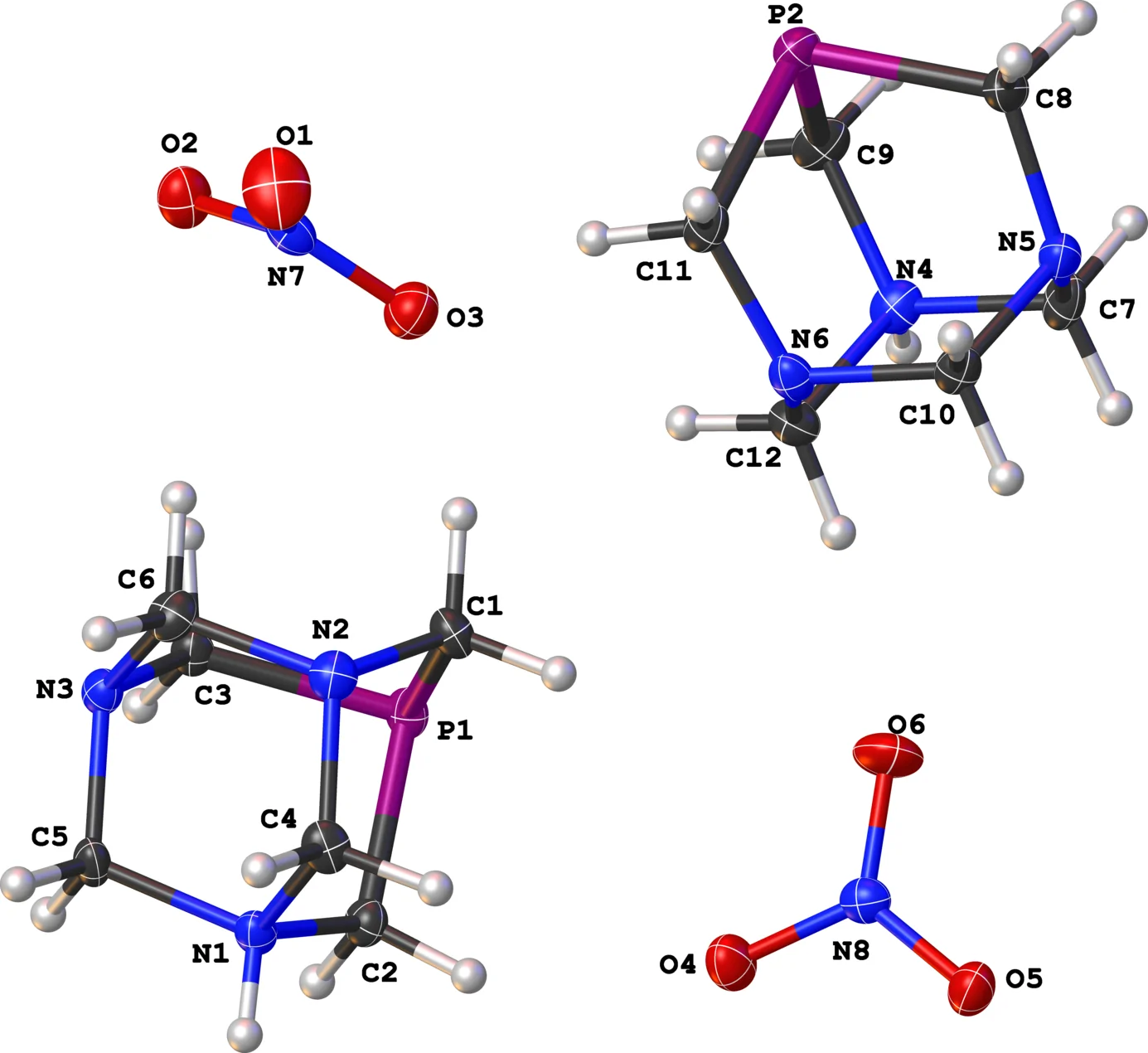
The mol­ecular structure of (**I**) shown with displacement ellipsoids at the 50% probability level. All C-bound hydrogen atoms are omitted for clarity.

**Figure 2:**
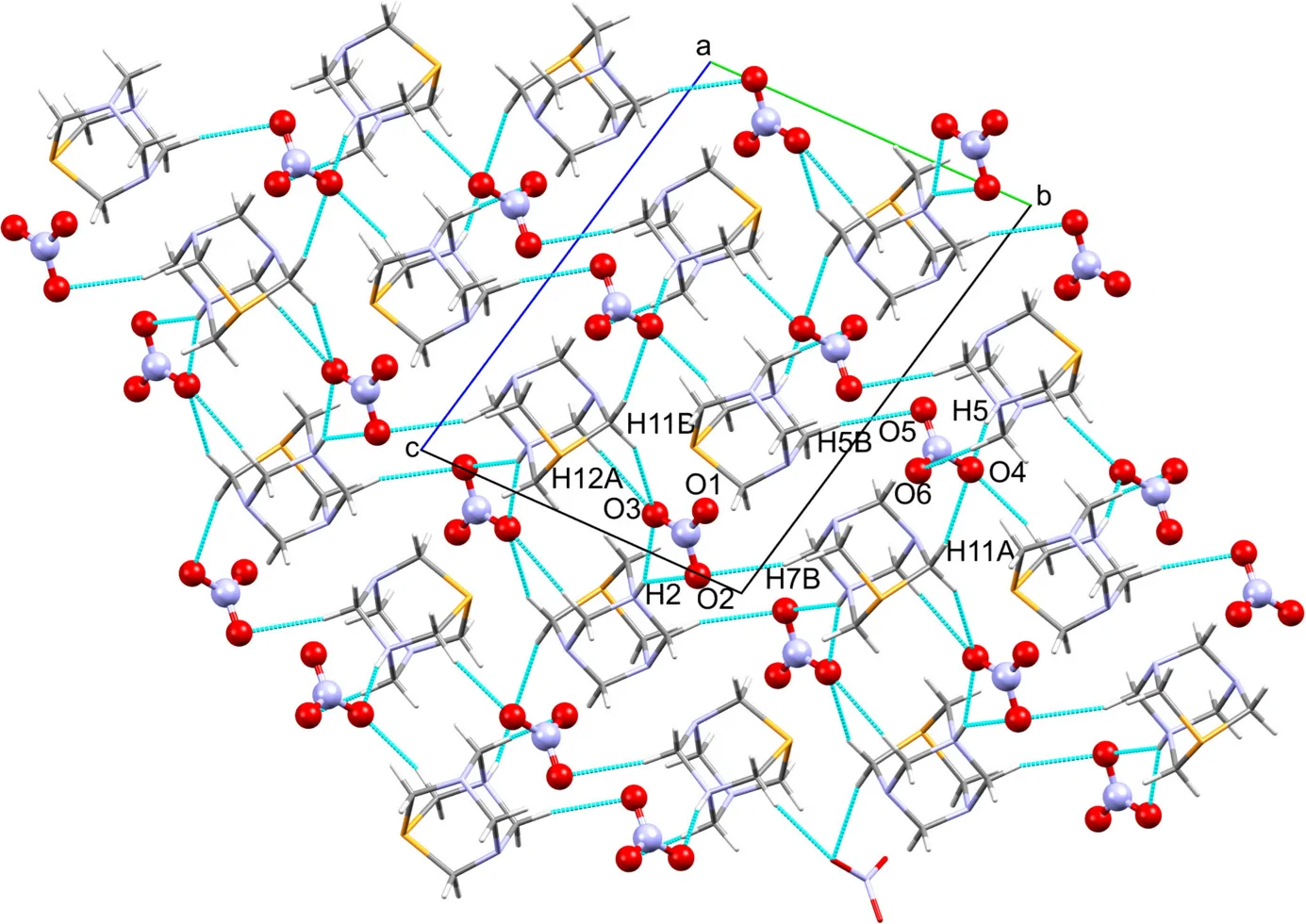
Representation of N—H⋯O and C—H⋯O hydrogen bonds in the crystal structure of (**I**) (cyan dotted bonds).

**Table 1:** Hydrogen-bond geometry (Å, °)

*D*—H⋯*A*	*D*—H	H⋯*A*	*D*⋯*A*	*D*—H⋯*A*
N1—H1⋯O4^i^	0.88 (3)	1.89 (3)	2.7576 (18)	166
N4—H4⋯O2^ii^	0.90 (3)	2.09 (3)	2.9076 (19)	151
N4—H4⋯O3^ii^	0.90 (3)	2.16 (3)	2.9448 (19)	145
C2—H2*B*⋯O4	0.99	2.30	3.202 (2)	151

Symmetry codes: (i) 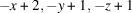; (ii) 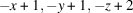.

**Table 2:** Experimental details

Crystal data	
Chemical formula	C_6_H_13_N_3_P^+^·NO_3_^−^
*M*_r_	220.17
Crystal system, space group	Triclinic, *P* 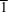
Temperature (K)	100
*a*, *b*, *c* (Å)	7.0750 (3), 9.8950 (4), 13.6434 (5)
α, β, γ (°)	102.614 (2), 90.123 (2), 90.613 (2)
*V* (Å^3^)	932.02 (6)
*Z*	4
Radiation type	Mo *K*α
μ (mm^−1^)	0.29
Crystal size (mm)	0.34 × 0.31 × 0.23

Data collection	
Diffractometer	Bruker APEXII CCD
Absorption correction	Multi-scan (*SADABS*; Krause *et al.*, 2015 ▸)
*T*_min_, *T*_max_	0.892, 0.937
No. of measured, independent and observed [*I* > 2σ(*I*)] reflections	14497, 4661, 4486
*R*_int_	0.024
(sin θ/λ)_max_ (Å^−1^)	0.676

Refinement	
*R*[*F*^2^ > 2σ(*F*^2^)], *wR*(*F*^2^), *S*	0.034, 0.100, 1.12
No. of reflections	4661
No. of parameters	262
H-atom treatment	H atoms treated by a mixture of independent and constrained refinement
Δρ_max_, Δρ_min_ (e Å^−3^)	0.58, −0.42

Computer programs: *APEX2*, *SAINT*, *SAINT-Plus* and *XPREP* (Bruker, 2008 ▸), *OLEX2.solve* (Bourhis *et al.*, 2015 ▸), *SHELXL2018/3* (Sheldrick, 2015 ▸) and *OLEX2* (Dolomanov *et al.*, 2009 ▸).

## full crystallographic data

### 1,3,5-Triaza-7-phosphaadamantan-1-ium nitrate Crystal data

**Table:**

C_6_H_13_N_3_P^+^·NO_3_^−^	*Z* = 4
*M**_r_* = 220.17	*F*(000) = 464
Triclinic, *P*1	*D*_x_ = 1.569 Mg m^−^^3^
*a* = 7.0750 (3) Å	Mo *K*α radiation, λ = 0.71073 Å
*b* = 9.8950 (4) Å	Cell parameters from 9920 reflections
*c* = 13.6434 (5) Å	θ = 2.9–28.7°
α = 102.614 (2)°	µ = 0.29 mm^−^^1^
β = 90.123 (2)°	*T* = 100 K
γ = 90.613 (2)°	Block, colourless
*V* = 932.02 (6) Å^3^	0.34 × 0.31 × 0.23 mm

### 1,3,5-Triaza-7-phosphaadamantan-1-ium nitrate Data collection

**Table:**

Bruker APEXII CCD diffractometer	4661 independent reflections
Graphite monochromator	4486 reflections with *I* > 2σ(*I*)
Detector resolution: 8 pixels mm^-1^	*R*_int_ = 0.024
φ and ω scans	θ_max_ = 28.7°, θ_min_ = 1.5°
Absorption correction: multi-scan (SADABS; Krause *et al.*, 2015)	*h* = −9→9
*T*_min_ = 0.892, *T*_max_ = 0.937	*k* = −13→11
14497 measured reflections	*l* = −18→18

### 1,3,5-Triaza-7-phosphaadamantan-1-ium nitrate Refinement

**Table:**

Refinement on *F*^2^	Primary atom site location: iterative
Least-squares matrix: full	Hydrogen site location: mixed
*R*[*F*^2^ > 2σ(*F*^2^)] = 0.034	H atoms treated by a mixture of independent and constrained refinement
*wR*(*F*^2^) = 0.100	*w* = 1/[σ^2^(*F*_o_^2^) + (0.0489*P*)^2^ + 0.5707*P*] where *P* = (*F*_o_^2^ + 2*F*_c_^2^)/3
*S* = 1.12	(Δ/σ)_max_ = 0.001
4661 reflections	Δρ_max_ = 0.58 e Å^−^^3^
262 parameters	Δρ_min_ = −0.42 e Å^−^^3^
0 restraints	

### 1,3,5-Triaza-7-phosphaadamantan-1-ium nitrate Special details

**Table:**

Experimental. School of Agriculture and Science, Discipline of Chemistry, University of KwaZulu-Natal, Private Bag X54001, Durban, 4000, South Africa
Geometry. All esds (except the esd in the dihedral angle between two l.s. planes) are estimated using the full covariance matrix. The cell esds are taken into account individually in the estimation of esds in distances, angles and torsion angles; correlations between esds in cell parameters are only used when they are defined by crystal symmetry. An approximate (isotropic) treatment of cell esds is used for estimating esds involving l.s. planes.
Refinement. Refined as a 2-component twin.

### 1,3,5-Triaza-7-phosphaadamantan-1-ium nitrate Fractional atomic coordinates and isotropic or equivalent isotropic displacement parameters (Å^2^)

**Table:**

	*x*	*y*	*z*	*U*_iso_*/*U*_eq_	
C1	0.5452 (2)	0.58160 (17)	0.67349 (12)	0.0177 (3)	
H1A	0.559898	0.484998	0.635389	0.021*	
H1B	0.430137	0.584724	0.715039	0.021*	
C2	0.9257 (2)	0.64444 (17)	0.65902 (11)	0.0159 (3)	
H2A	1.043130	0.688530	0.691149	0.019*	
H2B	0.957663	0.550475	0.620874	0.019*	
C3	0.7049 (2)	0.81794 (16)	0.79430 (11)	0.0151 (3)	
H3A	0.598675	0.832972	0.842440	0.018*	
H3B	0.817625	0.865805	0.829325	0.018*	
C4	0.6744 (2)	0.66343 (17)	0.53348 (11)	0.0171 (3)	
H4A	0.642100	0.712138	0.479655	0.020*	
H4B	0.697789	0.565164	0.501755	0.020*	
C5	0.8137 (2)	0.87607 (16)	0.64080 (11)	0.0159 (3)	
H5A	0.928007	0.916872	0.678346	0.019*	
H5B	0.783790	0.931556	0.590665	0.019*	
C6	0.4904 (2)	0.81704 (18)	0.65371 (12)	0.0186 (3)	
H6A	0.455189	0.871198	0.603550	0.022*	
H6B	0.383885	0.821402	0.701167	0.022*	
C7	0.3115 (3)	0.09677 (17)	0.85258 (12)	0.0202 (3)	
H7A	0.422824	0.046099	0.820145	0.024*	
H7B	0.267050	0.050265	0.905718	0.024*	
C8	−0.0136 (2)	0.14861 (17)	0.82629 (13)	0.0208 (3)	
H8A	−0.051385	0.095291	0.876739	0.025*	
H8B	−0.113529	0.134999	0.774059	0.025*	
C9	0.2147 (3)	0.32251 (19)	0.96221 (12)	0.0209 (3)	
H9A	0.260870	0.416957	0.993355	0.025*	
H9B	0.182965	0.275116	1.016978	0.025*	
C10	0.2283 (2)	0.16418 (17)	0.70205 (11)	0.0166 (3)	
H10A	0.126133	0.160151	0.651774	0.020*	
H10B	0.338778	0.115395	0.667117	0.020*	
C11	0.1177 (2)	0.39337 (17)	0.78380 (12)	0.0186 (3)	
H11A	0.024661	0.390929	0.729145	0.022*	
H11B	0.159792	0.490778	0.807723	0.022*	
C12	0.4297 (2)	0.31706 (18)	0.81655 (12)	0.0187 (3)	
H12A	0.461679	0.415170	0.846099	0.022*	
H12B	0.544255	0.272155	0.783434	0.022*	
N5	0.1644 (2)	0.09148 (14)	0.77929 (10)	0.0167 (3)	
N4	0.3693 (2)	0.24516 (15)	0.89982 (10)	0.0181 (3)	
N6	0.2811 (2)	0.31048 (14)	0.74263 (9)	0.0161 (3)	
N2	0.51768 (19)	0.67146 (15)	0.60160 (10)	0.0169 (3)	
N1	0.85319 (19)	0.72773 (14)	0.58714 (10)	0.0144 (2)	
N3	0.65804 (19)	0.88163 (14)	0.70931 (10)	0.0148 (3)	
N7	0.2859 (2)	0.76573 (15)	0.93218 (10)	0.0174 (3)	
N8	0.78748 (19)	0.20712 (15)	0.55432 (10)	0.0164 (3)	
O1	0.1566 (2)	0.77076 (17)	0.87169 (11)	0.0342 (3)	
O2	0.31980 (19)	0.86667 (14)	1.00409 (9)	0.0235 (3)	
O3	0.38752 (19)	0.65990 (14)	0.92249 (10)	0.0240 (3)	
O4	0.87968 (18)	0.32028 (13)	0.56299 (9)	0.0223 (3)	
O5	0.83586 (18)	0.10535 (13)	0.48843 (9)	0.0217 (3)	
O6	0.6530 (2)	0.19967 (16)	0.61060 (11)	0.0323 (3)	
P1	0.75278 (6)	0.62929 (4)	0.75817 (3)	0.01556 (10)	
P2	−0.00150 (6)	0.33549 (4)	0.88862 (3)	0.01797 (10)	
H1	0.941 (4)	0.728 (3)	0.5413 (18)	0.029 (6)*	
H4	0.471 (4)	0.243 (3)	0.9390 (19)	0.034 (7)*	

### 1,3,5-Triaza-7-phosphaadamantan-1-ium nitrate Atomic displacement parameters (Å^2^)

**Table:**

	*U*^11^	*U*^22^	*U*^33^	*U*^12^	*U*^13^	*U*^23^
C1	0.0194 (7)	0.0160 (7)	0.0186 (7)	−0.0037 (6)	−0.0006 (6)	0.0059 (6)
C2	0.0172 (7)	0.0147 (7)	0.0165 (7)	0.0023 (5)	−0.0009 (5)	0.0051 (5)
C3	0.0196 (7)	0.0144 (7)	0.0115 (6)	0.0003 (5)	0.0004 (5)	0.0032 (5)
C4	0.0208 (7)	0.0183 (8)	0.0120 (6)	−0.0022 (6)	−0.0039 (5)	0.0033 (5)
C5	0.0219 (7)	0.0111 (7)	0.0154 (6)	−0.0009 (5)	0.0022 (6)	0.0040 (5)
C6	0.0169 (7)	0.0191 (8)	0.0200 (7)	0.0036 (6)	−0.0029 (6)	0.0048 (6)
C7	0.0282 (8)	0.0148 (7)	0.0193 (7)	0.0062 (6)	0.0012 (6)	0.0072 (6)
C8	0.0197 (7)	0.0151 (7)	0.0257 (8)	−0.0020 (6)	0.0059 (6)	0.0002 (6)
C9	0.0258 (8)	0.0244 (8)	0.0118 (6)	0.0045 (7)	0.0010 (6)	0.0019 (6)
C10	0.0218 (7)	0.0163 (7)	0.0114 (6)	0.0026 (6)	0.0008 (5)	0.0023 (5)
C11	0.0215 (7)	0.0160 (7)	0.0193 (7)	0.0044 (6)	−0.0001 (6)	0.0057 (6)
C12	0.0167 (7)	0.0207 (8)	0.0194 (7)	−0.0014 (6)	0.0004 (6)	0.0060 (6)
N5	0.0193 (6)	0.0136 (6)	0.0174 (6)	0.0012 (5)	0.0029 (5)	0.0036 (5)
N4	0.0202 (7)	0.0209 (7)	0.0135 (6)	0.0041 (5)	−0.0034 (5)	0.0045 (5)
N6	0.0204 (6)	0.0162 (6)	0.0121 (5)	−0.0024 (5)	0.0010 (5)	0.0043 (5)
N2	0.0169 (6)	0.0179 (7)	0.0159 (6)	0.0002 (5)	−0.0032 (5)	0.0037 (5)
N1	0.0178 (6)	0.0137 (6)	0.0120 (5)	−0.0006 (5)	0.0017 (5)	0.0033 (5)
N3	0.0173 (6)	0.0133 (6)	0.0146 (6)	0.0019 (5)	0.0006 (5)	0.0049 (5)
N7	0.0176 (6)	0.0206 (7)	0.0158 (6)	−0.0032 (5)	−0.0009 (5)	0.0079 (5)
N8	0.0166 (6)	0.0200 (7)	0.0139 (6)	0.0022 (5)	−0.0001 (5)	0.0067 (5)
O1	0.0320 (7)	0.0357 (8)	0.0363 (8)	−0.0024 (6)	−0.0197 (6)	0.0108 (6)
O2	0.0307 (7)	0.0216 (6)	0.0174 (5)	−0.0017 (5)	−0.0020 (5)	0.0028 (5)
O3	0.0247 (6)	0.0220 (6)	0.0256 (6)	0.0031 (5)	0.0005 (5)	0.0057 (5)
O4	0.0259 (6)	0.0189 (6)	0.0207 (6)	−0.0038 (5)	0.0005 (5)	0.0014 (5)
O5	0.0263 (6)	0.0163 (6)	0.0219 (6)	0.0030 (5)	0.0035 (5)	0.0029 (5)
O6	0.0290 (7)	0.0378 (8)	0.0317 (7)	0.0015 (6)	0.0165 (6)	0.0113 (6)
P1	0.0199 (2)	0.01446 (19)	0.01372 (17)	0.00056 (15)	−0.00136 (14)	0.00611 (14)
P2	0.0180 (2)	0.0162 (2)	0.01841 (19)	0.00172 (15)	0.00339 (15)	0.00101 (15)

### 1,3,5-Triaza-7-phosphaadamantan-1-ium nitrate Geometric parameters (Å, º)

**Table:**

C1—H1A	0.9900	C8—H8A	0.9900
C1—H1B	0.9900	C8—H8B	0.9900
C1—N2	1.475 (2)	C8—N5	1.475 (2)
C1—P1	1.8583 (17)	C8—P2	1.8599 (17)
C2—H2A	0.9900	C9—H9A	0.9900
C2—H2B	0.9900	C9—H9B	0.9900
C2—N1	1.5046 (19)	C9—N4	1.498 (2)
C2—P1	1.8550 (16)	C9—P2	1.8488 (18)
C3—H3A	0.9900	C10—H10A	0.9900
C3—H3B	0.9900	C10—H10B	0.9900
C3—N3	1.4744 (19)	C10—N5	1.469 (2)
C3—P1	1.8587 (16)	C10—N6	1.476 (2)
C4—H4A	0.9900	C11—H11A	0.9900
C4—H4B	0.9900	C11—H11B	0.9900
C4—N2	1.440 (2)	C11—N6	1.465 (2)
C4—N1	1.522 (2)	C11—P2	1.8539 (17)
C5—H5A	0.9900	C12—H12A	0.9900
C5—H5B	0.9900	C12—H12B	0.9900
C5—N1	1.519 (2)	C12—N4	1.526 (2)
C5—N3	1.440 (2)	C12—N6	1.447 (2)
C6—H6A	0.9900	N4—H4	0.90 (3)
C6—H6B	0.9900	N1—H1	0.88 (3)
C6—N2	1.475 (2)	N7—O1	1.2395 (19)
C6—N3	1.469 (2)	N7—O2	1.2583 (19)
C7—H7A	0.9900	N7—O3	1.2598 (19)
C7—H7B	0.9900	N8—O4	1.2726 (19)
C7—N5	1.434 (2)	N8—O5	1.2461 (18)
C7—N4	1.520 (2)	N8—O6	1.2358 (18)
			
H1A—C1—H1B	107.6	H10A—C10—H10B	107.7
N2—C1—H1A	108.7	N5—C10—H10A	108.9
N2—C1—H1B	108.7	N5—C10—H10B	108.9
N2—C1—P1	114.41 (11)	N5—C10—N6	113.46 (12)
P1—C1—H1A	108.7	N6—C10—H10A	108.9
P1—C1—H1B	108.7	N6—C10—H10B	108.9
H2A—C2—H2B	107.8	H11A—C11—H11B	107.6
N1—C2—H2A	109.0	N6—C11—H11A	108.6
N1—C2—H2B	109.0	N6—C11—H11B	108.6
N1—C2—P1	112.97 (10)	N6—C11—P2	114.53 (11)
P1—C2—H2A	109.0	P2—C11—H11A	108.6
P1—C2—H2B	109.0	P2—C11—H11B	108.6
H3A—C3—H3B	107.6	H12A—C12—H12B	108.0
N3—C3—H3A	108.7	N4—C12—H12A	109.4
N3—C3—H3B	108.7	N4—C12—H12B	109.4
N3—C3—P1	114.36 (10)	N6—C12—H12A	109.4
P1—C3—H3A	108.7	N6—C12—H12B	109.4
P1—C3—H3B	108.7	N6—C12—N4	111.18 (13)
H4A—C4—H4B	108.0	C7—N5—C8	111.48 (13)
N2—C4—H4A	109.3	C7—N5—C10	109.32 (13)
N2—C4—H4B	109.3	C10—N5—C8	111.82 (13)
N2—C4—N1	111.63 (12)	C7—N4—C12	108.65 (12)
N1—C4—H4A	109.3	C7—N4—H4	108.4 (17)
N1—C4—H4B	109.3	C9—N4—C7	112.16 (14)
H5A—C5—H5B	108.0	C9—N4—C12	111.31 (13)
N1—C5—H5A	109.4	C9—N4—H4	108.9 (17)
N1—C5—H5B	109.4	C12—N4—H4	107.3 (17)
N3—C5—H5A	109.4	C11—N6—C10	111.89 (13)
N3—C5—H5B	109.4	C12—N6—C10	109.13 (13)
N3—C5—N1	111.00 (12)	C12—N6—C11	111.92 (12)
H6A—C6—H6B	107.7	C1—N2—C6	111.46 (12)
N2—C6—H6A	108.8	C4—N2—C1	111.54 (13)
N2—C6—H6B	108.8	C4—N2—C6	109.34 (13)
N3—C6—H6A	108.8	C2—N1—C4	111.58 (12)
N3—C6—H6B	108.8	C2—N1—C5	111.25 (12)
N3—C6—N2	113.82 (13)	C2—N1—H1	107.7 (16)
H7A—C7—H7B	108.0	C4—N1—H1	107.9 (16)
N5—C7—H7A	109.3	C5—N1—C4	109.21 (12)
N5—C7—H7B	109.3	C5—N1—H1	109.1 (17)
N5—C7—N4	111.52 (13)	C5—N3—C3	111.94 (12)
N4—C7—H7A	109.3	C5—N3—C6	109.19 (12)
N4—C7—H7B	109.3	C6—N3—C3	112.10 (13)
H8A—C8—H8B	107.6	O1—N7—O2	120.77 (15)
N5—C8—H8A	108.6	O1—N7—O3	120.72 (15)
N5—C8—H8B	108.6	O2—N7—O3	118.50 (14)
N5—C8—P2	114.68 (11)	O5—N8—O4	118.67 (13)
P2—C8—H8A	108.6	O6—N8—O4	120.04 (15)
P2—C8—H8B	108.6	O6—N8—O5	121.29 (15)
H9A—C9—H9B	107.8	C1—P1—C3	96.70 (7)
N4—C9—H9A	109.0	C2—P1—C1	96.64 (7)
N4—C9—H9B	109.0	C2—P1—C3	94.86 (7)
N4—C9—P2	112.85 (10)	C9—P2—C8	95.54 (8)
P2—C9—H9A	109.0	C9—P2—C11	96.54 (8)
P2—C9—H9B	109.0	C11—P2—C8	96.12 (8)
			
N5—C7—N4—C9	66.80 (17)	N1—C2—P1—C1	47.23 (12)
N5—C7—N4—C12	−56.67 (17)	N1—C2—P1—C3	−50.11 (12)
N5—C8—P2—C9	−50.15 (14)	N1—C4—N2—C1	−68.34 (17)
N5—C8—P2—C11	47.06 (14)	N1—C4—N2—C6	55.40 (16)
N5—C10—N6—C11	−66.12 (17)	N1—C5—N3—C3	67.37 (16)
N5—C10—N6—C12	58.29 (17)	N1—C5—N3—C6	−57.35 (16)
N4—C7—N5—C8	−66.85 (17)	N3—C3—P1—C1	−46.57 (12)
N4—C7—N5—C10	57.29 (16)	N3—C3—P1—C2	50.72 (12)
N4—C9—P2—C8	48.69 (14)	N3—C5—N1—C2	−67.05 (16)
N4—C9—P2—C11	−48.14 (14)	N3—C5—N1—C4	56.55 (16)
N4—C12—N6—C10	−56.64 (16)	N3—C6—N2—C1	66.20 (17)
N4—C12—N6—C11	67.76 (17)	N3—C6—N2—C4	−57.59 (17)
N6—C10—N5—C7	−58.57 (17)	P1—C1—N2—C4	62.84 (15)
N6—C10—N5—C8	65.37 (17)	P1—C1—N2—C6	−59.69 (15)
N6—C11—P2—C8	−47.61 (13)	P1—C2—N1—C4	−59.88 (14)
N6—C11—P2—C9	48.71 (13)	P1—C2—N1—C5	62.35 (15)
N6—C12—N4—C7	56.30 (17)	P1—C3—N3—C5	−64.15 (15)
N6—C12—N4—C9	−67.68 (17)	P1—C3—N3—C6	58.94 (15)
N2—C1—P1—C2	−48.54 (13)	P2—C8—N5—C7	63.47 (17)
N2—C1—P1—C3	47.17 (12)	P2—C8—N5—C10	−59.25 (16)
N2—C4—N1—C2	67.66 (16)	P2—C9—N4—C7	−60.94 (16)
N2—C4—N1—C5	−55.74 (16)	P2—C9—N4—C12	61.03 (16)
N2—C6—N3—C3	−65.97 (17)	P2—C11—N6—C10	60.33 (15)
N2—C6—N3—C5	58.66 (17)	P2—C11—N6—C12	−62.51 (16)

### 1,3,5-Triaza-7-phosphaadamantan-1-ium nitrate Hydrogen-bond geometry (Å, º)

**Table:**

*D*—H···*A*	*D*—H	H···*A*	*D*···*A*	*D*—H···*A*
N1—H1···O4^i^	0.88 (3)	1.89 (3)	2.7576 (18)	166
N4—H4···O2^ii^	0.90 (3)	2.09 (3)	2.9076 (19)	151
N4—H4···O3^ii^	0.90 (3)	2.16 (3)	2.9448 (19)	145
C2—H2*B*···O4	0.99	2.30	3.202 (2)	151

Symmetry codes: (i) −*x*+2, −*y*+1, −*z*+1; (ii) −*x*+1, −*y*+1, −*z*+2.

## References

[bb1] Akbayeva, D. N., Moneti, S., Peruzzini, M., Gonsalvi, L., Ienco, A. & Vizza, F. (2005). *C. R. Chim.* **8**, 1491–1496.

[bb9] Bourhis, L. J., Dolomanov, O. V., Gildea, R. J., Howard, J. A. K. & Puschmann, H. (2015). *Acta Cryst.* A**71**, 59–75. 10.1107/S2053273314022207 PMC4283469 25537389

[bb2] Bruker (2014). *APEX2* and *SAINT*. Bruker AXS Inc., Madison, Wisconsin, USA.

[bb3] Daigle, D. J., Poulain, N., Harvey, P. D. & Bhatt, S. (1998). *Inorg. Synth.* **32**, 40–45.

[bb4] Dolomanov, O. V., Bourhis, L. J., Gildea, R. J., Howard, J. A. K. & Puschmann, H. (2009). *J. Appl. Cryst.* **42**, 339–341.

[bb5] Kirillov, A. M., Smoleński, P., Guedes da Silva, M. F. C. & Pombeiro, A. J. (2007). *Eur. J. Inorg. Chem.* pp. 2686–2692.

[bb6] Krause, L., Herbst-Irmer, R., Sheldrick, G. M. & Stalke, D. (2015). *J. Appl. Cryst.* **48**, 3–10. 10.1107/S1600576714022985 PMC4453166 26089746

[bb7] Sheldrick, G. M. (2015). *Acta Cryst.* C**71**, 3–8.

[bb8] Tu, X., Nichol, G. S., Wang, R. & Zheng, Z. (2008). *Dalton Trans.* pp. 6030–6038. 10.1039/b806888g 19082060

